# Etiology of Severe Non-malaria Febrile Illness in Northern Tanzania: A Prospective Cohort Study

**DOI:** 10.1371/journal.pntd.0002324

**Published:** 2013-07-18

**Authors:** John A. Crump, Anne B. Morrissey, William L. Nicholson, Robert F. Massung, Robyn A. Stoddard, Renee L. Galloway, Eng Eong Ooi, Venance P. Maro, Wilbrod Saganda, Grace D. Kinabo, Charles Muiruri, John A. Bartlett

**Affiliations:** 1 Division of Infectious Diseases and International Health, Department of Medicine, Duke University Medical Center, Durham, North Carolina, United States of America; 2 Department of Pathology, Duke University Medical Center, Durham, North Carolina, United States of America; 3 Duke Global Health Institute, Duke University, Durham, North Carolina, United States of America; 4 Kilimanjaro Christian Medical Centre, Moshi, Tanzania; 5 Kilimanjaro Christian Medical College, Tumaini University, Moshi, Tanzania; 6 Centre for International Health, Dunedin School of Medicine, University of Otago, Dunedin, New Zealand; 7 Rickettsial Zoonoses Branch, Division of Vector-Borne Diseases, National Center for Emerging and Zoonotic Infectious Diseases, Centers for Disease Control and Prevention, Atlanta, Georgia, United States of America; 8 Bacterial Special Pathogens Branch, Division of High-Consequence Pathogens and Pathology, National Center for Emerging and Zoonotic Infectious Diseases, Centers for Disease Control and Prevention, Atlanta, Georgia, United States of America; 9 Emerging Infectious Diseases Signature Research Program, Duke-National University of Singapore Graduate Medical School, Singapore, Singapore; 10 Mawenzi Regional Hospital, Moshi, Tanzania; Institut Pasteur, France

## Abstract

**Introduction:**

The syndrome of fever is a commonly presenting complaint among persons seeking healthcare in low-resource areas, yet the public health community has not approached fever in a comprehensive manner. In many areas, malaria is over-diagnosed, and patients without malaria have poor outcomes.

**Methods and Findings:**

We prospectively studied a cohort of 870 pediatric and adult febrile admissions to two hospitals in northern Tanzania over the period of one year using conventional standard diagnostic tests to establish fever etiology. Malaria was the clinical diagnosis for 528 (60.7%), but was the actual cause of fever in only 14 (1.6%). By contrast, bacterial, mycobacterial, and fungal bloodstream infections accounted for 85 (9.8%), 14 (1.6%), and 25 (2.9%) febrile admissions, respectively. Acute bacterial zoonoses were identified among 118 (26.2%) of febrile admissions; 16 (13.6%) had brucellosis, 40 (33.9%) leptospirosis, 24 (20.3%) had Q fever, 36 (30.5%) had spotted fever group rickettsioses, and 2 (1.8%) had typhus group rickettsioses. In addition, 55 (7.9%) participants had a confirmed acute arbovirus infection, all due to chikungunya. No patient had a bacterial zoonosis or an arbovirus infection included in the admission differential diagnosis.

**Conclusions:**

Malaria was uncommon and over-diagnosed, whereas invasive infections were underappreciated. Bacterial zoonoses and arbovirus infections were highly prevalent yet overlooked. An integrated approach to the syndrome of fever in resource-limited areas is needed to improve patient outcomes and to rationally target disease control efforts.

## Author Summary

The syndrome of fever is caused by a large number of infectious diseases. Malaria is thought to have been declining in the tropics since 2004. Increasing use of malaria diagnostic tests reveal a growing proportion of patients with fever who do not have malaria. While malaria diagnostic tests may be available, healthcare workers have few tools to diagnose causes of fever other than malaria. In order to identify major causes of fever other than malaria in northern Tanzania, we studied 870 patients with fever who were sufficiently ill to require admission to hospital. Malaria was uncommon and over-diagnosed, whereas invasive infections, including bloodstream infections, were underappreciated. Infections associated with animals such as brucellosis, leptospirosis, Q fever, and spotted fever group rickettsioses as well as viral infections transmitted by mosquitoes were common yet overlooked. We recommend that research on the syndrome of fever in resource-limited areas should focus on a wide range of potential causes. Animal-associated infections should be prioritized in patient management and disease control.

## Introduction

Fever without a localized cause is one of the most common presenting complaints among persons seeking healthcare in many low- and middle-income countries [1], [2]. However, unlike the syndromes of pneumonia and diarrhea that feature in global disease burden estimates and have well coordinated programs integrating efforts across the range of responsible pathogens to avert morbidity and mortality, there has been a lack of a coordinated approach for febrile illness. While illness and death due to some specific infections causing fever, such as malaria [3] and increasingly bacterial sepsis are well quantified [4]–[6], others such as a range of zoonoses and viral infections are uncounted and consequently may be underappreciated.

The various etiologies of febrile illnesses are difficult to distinguish from one another clinically [7], [8]. As clinical laboratory services are often limited in areas where febrile conditions are particularly common [9], [10], clinicians may have few diagnostic tools to establish an etiologic diagnosis. Therefore, clinical management is often driven by syndrome-based guidelines employing empiric treatment [11]–[13]. In the absence of systematically collected data on fever etiology, considerable mismatch between clinical diagnosis, clinical management, and actual etiology may occur resulting in poor patient outcomes [14]. It is increasingly recognized that malaria is over-diagnosed in many areas [14], [15]. To address this problem, the World Health Organization (WHO) malaria treatment guidelines moved away from clinical diagnosis of malaria to treatment based on the results of a malaria diagnostic test such as a blood smear or a malaria rapid diagnostic test. With more widespread availability of diagnostic tests to exclude malaria and apparent declines in malaria worldwide [3], clinicians in resource-limited areas are faced with a growing proportion of febrile patients who do not have malaria and few tools to guide subsequent management.

We sought to describe comprehensively the causes of febrile illness in northern Tanzania among patients sufficiently ill to require hospitalization. Febrile patients admitted to two hospitals were evaluated for a wide range of infectious etiologies using conventional standard diagnostic techniques.

## Methods

### Ethics statement

This study was approved by the Kilimanjaro Christian Medical Centre (KCMC) Research Ethics Committee, the Tanzania National Institutes for Medical Research National Research Ethics Coordinating Committee, and Institutional Review Boards of Duke University Medical Center and the CDC. All minors had written informed consent given from a parent or guardian and all adult participants provided their own written informed consent.

### Setting

Moshi (population, >144 000) is the administrative center of the Kilimanjaro Region (population, >1.4 million) in northern Tanzania and is situated at an elevation of 890 m above mean sea level. The climate is characterized by a long rainy period (March–May) and a short rainy period (October–December) [16]. Malaria transmission intensity is low [17]. KCMC is a consultant referral hospital with 458 inpatient beds serving several regions in northern Tanzania, and Mawenzi Regional Hospital (MRH), with 300 beds, is the Kilimanjaro Regional hospital. Together KCMC and MRH serve as the main providers of hospital care in the Moshi area. In 2008, KCMC admitted 22,099 patients and MRH admitted 21,763 patients.

### Study design

A study team that was independent of the hospital clinical team identified participants among infants and children admitted to KCMC from 17 September 2007 through 25 August 2008, and among adolescents and adult admitted to KCMC and MRH in Moshi, Tanzania, from 17 September 2007 through 31 August 2008. The methods of these studies have been described in detail elsewhere [7], [8]. In brief, all admitted patients were screened for eligibility by study team members as soon as possible after admission and no later than 24 hours after admission. Infants and children aged from ≥2 months to <13 years, with a history of fever in the past 48 h or an axillary temperature ≥37.5°C or a rectal temperature of ≥38.0°C, and adolescents and adults aged ≥13 years and with oral temperatures of ≥38.0°C were invited to participate in the study. Patients admitted with known malignancy, renal failure, hepatic failure, bone marrow aplasia, trauma or surgery were excluded.

A standardized clinical history and physical examination were performed on consenting patients by a trained clinical officer who was a member of the study team and who worked in parallel with the hospital admitting team. Provisional diagnoses by the hospital clinical team made independently of the study team were recorded and coded using the International Statistical Classification of Diseases and Related Health Problems, 10th Revision (ICD-10) codes. Following cleansing of the skin with isopropyl alcohol and povidone iodine, blood was drawn from adults and adolescents for aerobic blood culture (5 mL) and for mycobacterial blood culture (5 mL) and from pediatric patients for a single aerobic blood culture (4 ml). In addition, blood was drawn for complete blood count, examination for blood parasites, and HIV antibody testing. Acute serum, plasma, and whole blood were archived on all participants. For patients found to be HIV seropositive, CD4-positive T lymphocyte count (CD4 cell count) and serum cryptococcal antigen level were also measured. HIV-seronegative patients were screened for the presence of acute HIV infection by polymerase chain reaction (PCR) for HIV-1 RNA. Urine was collected as soon as possible after admission for detection of urine antimicrobial activity and for antigen detection. A discharge form was completed at the time of discharge from the hospital that captured whether the patient died in hospital, the in-hospital management, and the discharge diagnoses coded using ICD-10 codes. The results of study investigations done in Moshi were provided immediately to the hospital clinical team to inform patient management. The results of investigations done at reference laboratories were provided to the hospital clinical team as they became available. The hospital clinical team was responsible for all aspects of patient management, following clinical judgment and use of locally adapted and developed treatment guidelines. All participants were asked to return to a study clinic 4–6 weeks after enrollment to provide a convalescent serum sample. To promote high levels of follow up, the study team provided a follow up appointment card prior to hospital discharge, made reminder telephone calls to participants during the week prior to the appointment, reimbursed travel expenses of returning participants, and when necessary a field worker made home visits.

### Laboratory evaluations

Laboratory evaluations were selected to reflect a range of infectious diseases that might occur in northern Tanzania. Priority was given to laboratory evaluations for infectious diseases that might require specific management.

#### Malaria

Thick and thin blood films stained with Giemsa were examined for blood parasites by oil immersion microscopy. Parasite density was determined by standard methods [18].

#### Bacteria and fungal bloodstream infections

Blood culture bottles were assessed for volume adequacy comparing the weight before and after inoculation with blood. Adequate volume was defined as the recommended volume ±20%. BacT/ALERT standard aerobic and mycobacterial bottles were loaded into the BacT/ALERT 3D Microbial Detection system (BioMérieux), where they were incubated for 5 and 42 days, respectively. Standard methods were used for identifying bloodstream isolates [7], [8].

#### Serum antigen testing

Cryptococcal antigen level was measured using the Latex Cryptococcal Antigen Detection System assay (Immuno-Mycologics).

#### Urine antigen testing

Urine was tested for all participants for *Legionella pneumophila* serogroup 1 antigen using the Binax NOW *Legionella* urinary antigen test, and for adolescents and adults using the *Sreptococcus pneumoniae* using the Binax NOW *S. pneumoniae* antigen test (Binax). Urine was tested for *Histoplasma capsulatum* antigen using the MVista *H. capsulatum* quantitative antigen enzyme immunoassay (Miravista Diagnostics) [19], [20].

#### Leptospirosis

Leptospirosis laboratory diagnosis was made using the standard microscopic agglutination test (MAT) performed at the CDC. Live leptospiral cell suspensions representing 20 serovars and 17 serogroups described elsewhere [21] were incubated with serially diluted serum specimens. Resulting agglutination titers were read using darkfield microscopy. The reported titer was the highest dilution of serum that agglutinated at least 50% of the cells for each serovar tested [22]. Confirmed leptospirosis was defined as a ≥4-fold rise in the agglutination titer between acute and convalescent serum samples [23].

#### Brucellosis

Brucellosis serology was performed using the standard microagglutination test (MAT) performed at the CDC. Standardized *Brucella abortus* strain 1119-3 killed antigen (National Veterinary Services Laboratory, Ames, IA) was used for MAT at a 1∶25 working dilution described elsewhere [24]. Results were read on a Scienceware Plate Reader (Bel-Art Products, Wayne, NJ). Minor modifications were made to the CDC's standard MAT, including the use of U-bottom plates, incubation at 26°C, and discontinued use of staining techniques [25]. Confirmed brucellosis was defined as a ≥4-fold rise in the agglutination titer between acute and convalescent serum samples.

#### Q fever

Convalescent-phase serum samples were screened using *C. burnetii* immunoglobulin (Ig) G enzyme-linked immunosorbent assay (ELISA) against Phase II antigen (Inverness Medical Innovations). For samples that were either positive or equivocal by ELISA, paired serum samples were tested by indirect immunofluorescence antibody (IFA) IgG assay to *C. burnetii* (Nine Mile strain) Phase I and Phase II antigens. A fourfold or greater increase in IFA reciprocal titer to Phase II antigen defined acute Q fever [26].

#### Spotted fever group and typhus group rickettsioses

Serum samples were tested for SFGR and TGR by IgG IFA to *R. conorii* (Moroccan strain) and to *R. typhi* (Wilmington strain), respectively. Among paired serum samples, a fourfold or greater increase in IFA titer to *R. conorii* and *R. typhi* defined acute SFGR and TGR infections, respectively [26].

#### Arboviruses

RNA was extracted from serum samples using the QIAamp Viral RNA Mini kit (QIAGEN, Hilden, Germany). Reverse transcription was performed using Invitrogen Superscript III First Strand Synthesis System (Life Technologies, Carlsbad, CA). Real-time PCRs for flavivirus, DENV, and CHIKV were carried out with the LightCycler 480 SYBR Green I Master kit (Roche Diagnostics, Penzberg, Germany) in a total reaction volume of 20 µL containing 2 µL of cDNA using primers published elsewhere [27]–[29]. Confirmed acute CHIKV, DENV, and flavivirus infections were defined as a positive PCR result for CHIKV, DENV, and flavivirus viral RNA, respectively [30].

#### HIV

HIV-1 antibody testing was done on whole blood using both the Capillus HIV-1/HIV-2 (Trinity Biotech) and Determine HIV-1/HIV-2 (Abbott Laboratories) rapid HIV antibody tests. The Capillus test was replaced with the SD Bioline HIV-1/HIV-2 test (version 3.0; Standard Diagnostics) on 4 March 2008 after a change in Tanzania Ministry of Health HIV testing guidelines. If rapid tests were discordant, the sample was tested using enzyme-linked immunosorbent assay (ELISA; Vironostika Uni-Form II plus O Ab; bioMe'rieux). If the ELISA was negative, no further testing was done. If the ELISA was positive, a Western blot (Genetic Systems HIV-1 Western blot kit; Bio-Rad) was done to confirm the result [31]. HIV-1 RNA PCR was done using the Abbott m2000 system RealTime HIV-1 assay (Abbott Laboratories) [32], [33].

### Statistic analysis

Data were entered using the Cardiff Teleform system (Cardiff Inc., Vista, Ca., USA) into an Access database (Microsoft Corp, Va., USA). When a diagnostic test was not applied to the whole cohort due lack of availability of an acute or convalescent sample, the proportion of confirmed cases in the tested group was extrapolated to the untested group by assuming that prevalence was the same in the tested group as in the untested group. Statistical analyses were performed with SAS version 9.1 software (SAS Inc, Cary, NC).

## Results

### Participant characteristics

**Figure 1:**
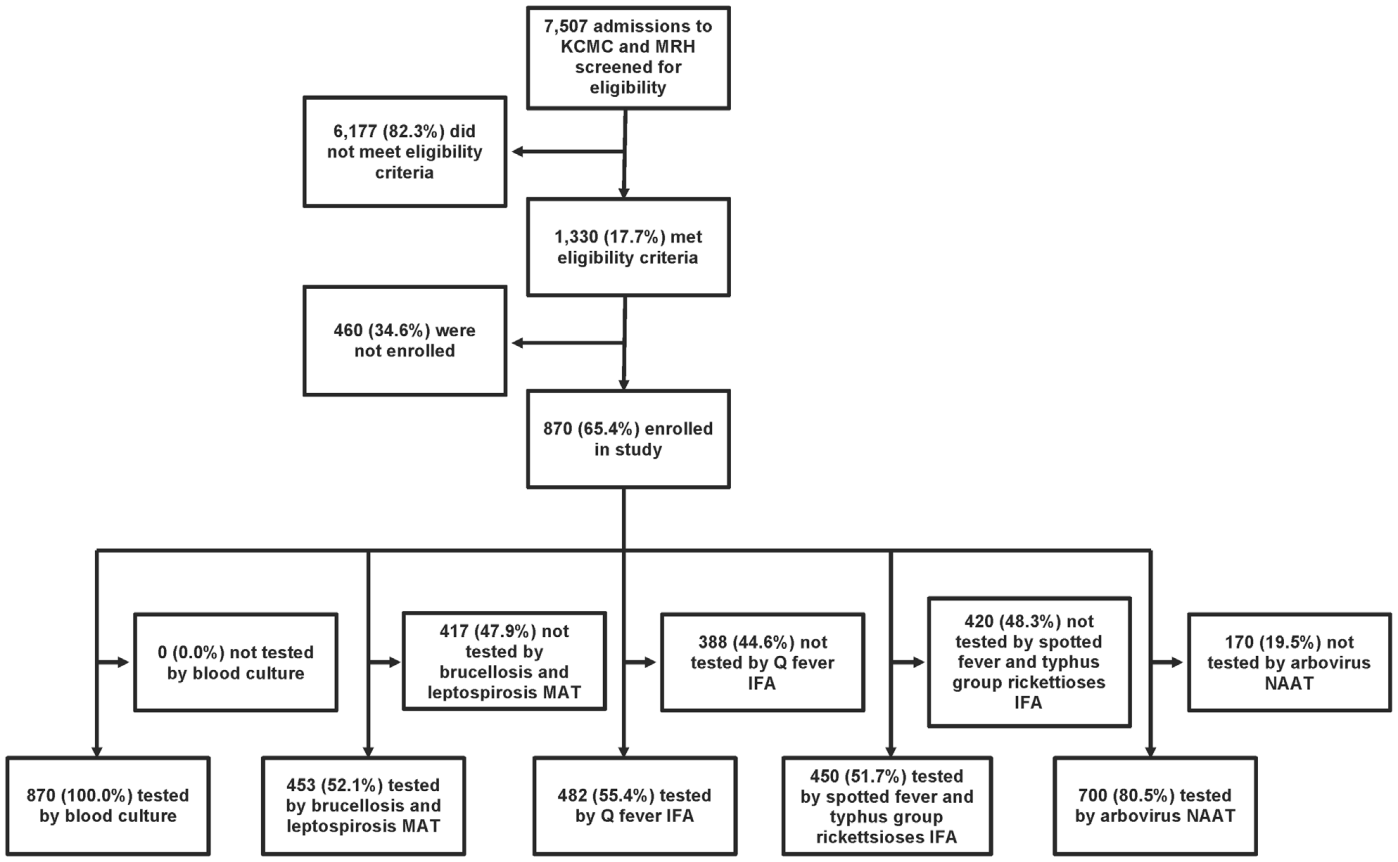
Study flow diagram. KCMC: Kilimanjaro Christian Medical Centre; MRH: Mawenzi Regional Hospital; MAT: microagglutination test; IFA: immunoflouresence assay; NAAT: nucleic acid amplification test.

Figure 1 summarizes participant screening, enrollment, and diagnostic testing. Of 870 febrile admissions to two hospitals in northern Tanzania enrolled in the study 484 (55.6%) were female. Of participants, 467 (53.7%) were infants and children with a median (range) age of 2 years (2 months - 13 years); the remainder adolescents and adults with a median (range) age of 38 (14–96) years. Fifty seven (12.2%) infants and children were HIV-infected compared with 157 (39.0%) adolescents and adults. Among infants and children 34 (7.3%) of 464 with hospital outcome data died; 2 (5.9%) of those who died had invasive infections. Among adolescents and adults, 41 (10.3%) of 398 with hospital outcome data died; 11 (26.8%) of those who died had invasive infections. In hospital deaths could not be attributed to etiologies requiring serologic diagnosis due to the requirement for testing a convalescent serum sample.

### Proportions of febrile admissions attributed to specific etiologies

Table 1 shows the number of patients with acute and convalescent samples available for testing for each etiologic agent or group of etiologic agents. Not all tests could be applied to all participants because of limited volumes of sample for some participants, and by the lack of availability of convalescent serum for participants who died before the follow up visit or who did not return. The number of confirmed cases in each group is also shown. The proportion of febrile admissions attributed to each etiology is calculated. A complete sample set was available for 243–467 (52.0–100.0%) infants and children and for 207–403 (51.4–100.0%) adolescents and adults.

**Table 1:** Calculation of the proportion of hospitalized infants and children, and adolescents and adults, with specific etiologies of febrile illness, northern Tanzania, 2007–8.

Etiology	Infants and children			Adults and adolescents			All		
	n confirmed cases	n tested	(%)	n confirmed cases	n tested	(%)	n confirmed cases	n tested	(%)
Bloodstream infections									
Bacterial	16	467	(3.4)	69	403	(17.1)	85	870	(9.8)
Mycobacterial	0	467	(0.0)	14	403	(3.5)	14	870	(1.6)
Fungal	4	467	(0.9)	21	403	(5.2)	25	870	(2.9)
Malaria	6	467	(1.3)	8	403	(2.0)	14	870	(1.6)
Subtotal	26	467	(5.6)	112	403	(27.8)	138	870	(15.9)
Bacterial zoonoses									
Brucellosis	5	246	(2.0)	11	207	(5.3)	16	453	(3.5)
Leptospirosis	19	246	(7.7)	21	207	(10.1)	40	453	(8.8)
Q fever	7	268	(2.6)	17	215	(7.9)	24	482	(5.0)
Spotted fever group rickettsioses	18	243	(7.4)	18	207	(8.7)	36	450	(8.0)
Typhus group rickettsioses	0	243	(0.0)	2	207	(1.0)	2	450	(0.4)
Subtotal	49	243	(20.2)	69	207	(33.3)	118	450	(26.2)
Arboviruses									
Chikungunya	34	332	(10.2)	21	368	(5.7)	55	700	(7.9)
Flaviviruses	0	332	(0.0)	0	368	(0.0)	0	700	(0.0)
Subtotal	34	332	(10.2)	21	368	(5.7)	55	700	(7.9)
No diagnosis			(64.0)			(33.2)			(50.1)

Due to changing denominators for individual diagnostic tests, the proportion with no diagnosis is calculated as the proportion without a positive result from any test. Bloodstream infections are those diagnosed predominantly by blood culture, including organisms such as *Salmonella enterica*, *Streptococcus pneumoniae*, *Cryptococcus neoformans*, and *Mycobacterium tuberculosis*. Bacterial zoonoses, including brucellosis, leptospirosis, Q fever, and rickettsioses were diagnosed predominantly by serology, based on a 4-fold or greater rise in antibody titer between an acute and convalescent sample.

### Etiology of fever among infants and children

Of 467 infants and children enrolled, malaria was the clinical diagnosis for 282 (60.4%), but was the actual cause of fever in 6 (1.3%). Bacterial and fungal bloodstream infections described in detail elsewhere [8] accounted for 16 (3.4%) and 4 (0.9%) febrile admissions, respectively, and were underrepresented on admission differential diagnoses. Bacterial zoonoses were identified among 49 (20.2%) of febrile admissions; 5 (2.0%) had brucellosis, 19 (7.7%) leptospirosis, 7 (2.6%) had Q fever, 18 (7.4%) had spotted fever group rickettsioses, and none had typhus group rickettsioses. In addition, 34 (10.2%) of participants had a confirmed acute arbovirus infection, all due to chikungunya (Table 1). No patient had a bacterial zoonoses or an arbovirus infection included in the admission differential diagnosis.

### Etiology of fever among adolescents and adults

Of 403 adolescents and adults enrolled, malaria was the clinical diagnosis for 254 (63.0%), but was the actual cause of fever in 8 (2.0%). Bacterial, mycobacterial, and fungal bloodstream infections described in detail elsewhere [7] accounted for 69 (17.1%), 14 (3.5%), and 21 (5.2%) febrile admissions, respectively, and were underrepresented on admission differential diagnoses. Bacterial zoonoses were identified among 69 (33.3%) of febrile admissions; 11 (5.3%) had brucellosis, 21 (10.1%) leptospirosis, 17 (7.9%) had Q fever, 18 (8.7%) had spotted fever group rickettsioses, and 2 (1.0%) had typhus group rickettsioses. In addition, 21 (5.7%) of participants had a confirmed acute arbovirus infection, all due to chikungunya (Table 1). No patient had a bacterial zoonosis or an arbovirus infection included in the admission differential diagnosis.

### Etiology of fever overall

**Figure 2:**
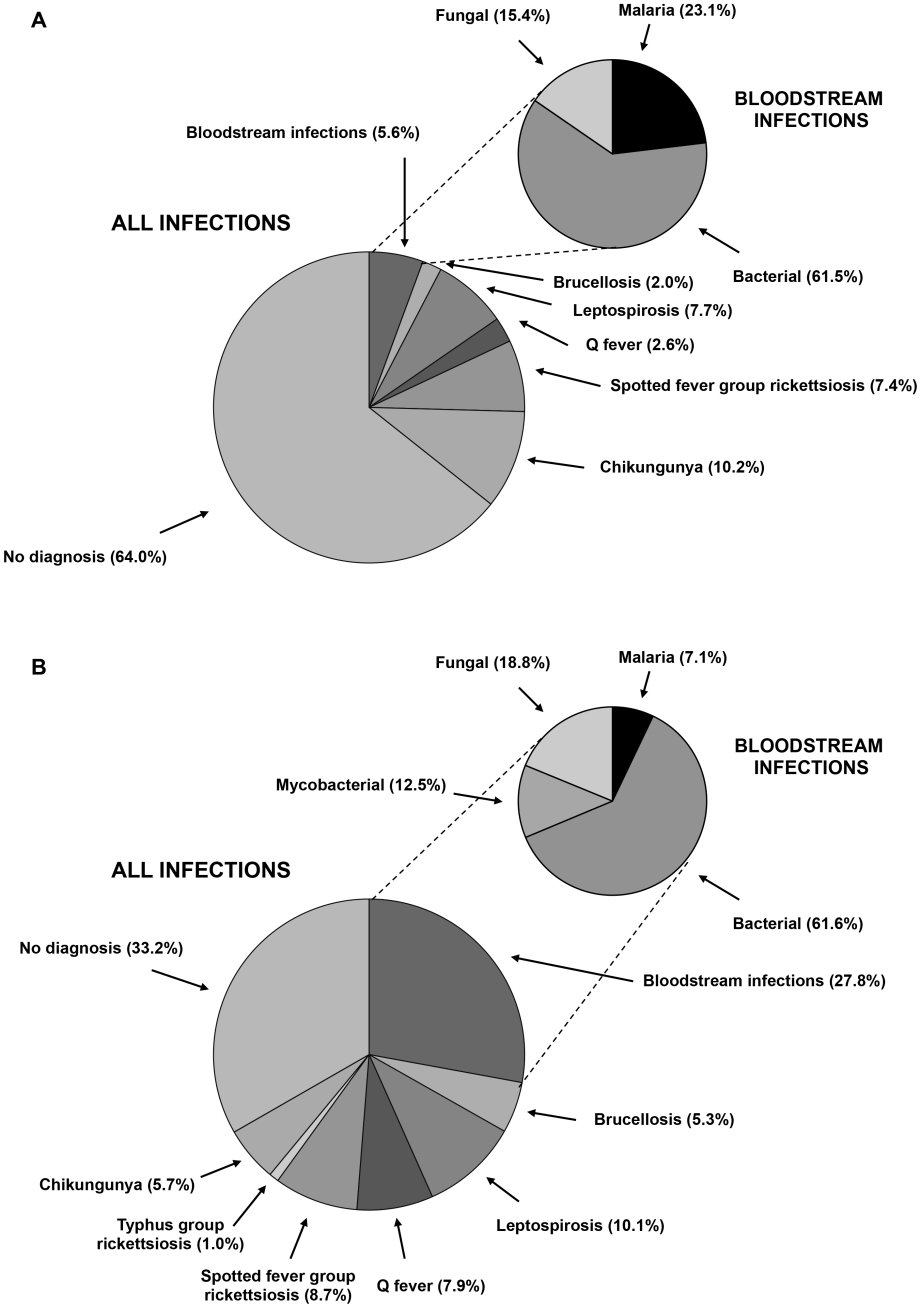
Laboratory confirmed causes of febrile illness among infants and children (panel A) and adolescents and adults (panel B) hospitalized in northern Tanzania, 2007–8*. *In instances that diagnostic test results were not available for all participants, the proportion positive from Table 1 was applied to the whole study population. Pie graphs do not account for concurrent infections. A complete listing of specific bacterial, mycobacterial, and fungal bloodstream infections is available elsewhere [7], [8].

Among all 870 participants, malaria was the clinical diagnosis for 528 (60.7%), but was the actual cause of fever in 14 (1.6%). By contrast, bacterial, mycobacterial, and fungal bloodstream infections accounted for 85 (9.8%), 14 (1.6%), and 25 (2.9%) febrile admissions, respectively, and were underrepresented on admission differential diagnoses. Bacterial zoonoses were identified among 118 (26.2%) of febrile admissions; 16 (13.6%) had brucellosis, 40 (33.9%) leptospirosis, 24 (20.3%) had Q fever, 36 (30.5%) had spotted fever group rickettsioses, and 2 (1.8%) had typhus group rickettsioses. In addition, 55 (7.9%) of participants had a confirmed acute arbovirus infection, all due to chikungunya (Table 1). No patient had a bacterial zoonoses or an arbovirus infection included in the admission differential diagnosis. The proportional etiology of febrile illness among study participants after extrapolating to the untested group is summarized in Figure 2.

## Discussion

We demonstrate among hospitalized febrile patients in northern Tanzania that malaria is uncommon and over-diagnosed, while invasive bacterial, mycobacterial, and fungal infections are underappreciated. At the same time, the bacterial zoonoses leptospirosis, Q fever, and spotted fever rickettsioses, and to a lesser extent brucellosis, and the arbovirus infection chikungunya are common yet unrecognized causes of fever. Our findings point to important mismatches between clinical diagnosis and management with actual diagnoses that have major implications for patient care, disease control and prevention, and for judicious use of antimalarial medications.

While the problem of malaria over-diagnosis has been appreciated for some time [14], [15], studies that comprehensively describe the causes of severe non-malaria fever requiring hospital admission beyond bloodstream infections have been lacking. The over-diagnosis of malaria results in inappropriate use of antimalarial medications and may be associated with higher case fatality rates among patients treated for malaria who do not have the infection [14], [15], [34]. While the underlying causes of the over-diagnosis of malaria are complex [35], the lack of epidemiologic information about the importance of alternative infections and guidance on their management is likely to play a role. Our findings confirm the potential benefits of making reliable malaria diagnostic tests available at healthcare facilities and using the results as the basis for prescription of antimalarial medications [36]. When adopted, such an approach to malaria treatment would support the judicious use of antimalarials and would define the population of patients with nonmalaria fever.

We found that the bacterial zoonoses, leptospirosis, Q fever, and spotted fever group rickettsioses, and to a lesser extent brucellosis, are major causes of febrile illness among patients sufficiently unwell to require hospitalization. That a group of neglected bacterial zoonoses are of major clinical and public health importance in sub-Saharan Africa is a new and paradigm-changing finding. For clinical practice, with the exception of leptospirosis that may be effectively treated with commonly prescribed antibacterials, patients with brucellosis, Q fever, and the rickettsioses are likely to leave hospital without specific treatment. In northern Tanzania where many rely on livestock for their health and economic wellbeing, *Leptospira*, *Brucella*, and *Coxiella* spp. also indirectly affect human health through their impact on animal fertility, growth, and survival. The control and prevention of the neglected bacterial zoonoses is likely to involve interventions that require the collaboration of human health experts with the animal and environmental health disciplines, an approach that is underdeveloped in many parts of the world.

Clinical guidelines for management of febrile patients in low resource areas focus on the identification and treatment of malaria and bacterial sepsis [11]–[13]. Our findings suggest that there is a need to identify and incorporate guidance on when to use a tetracycline for treatment of Q fever or rickettsial infection and when to consider treatment for brucellosis. We have previously demonstrated that features of the clinical history and physical examination do not perform well for identifying fever etiology [7], [8], [21], [26], [30]. Therefore, improvements to treatment algorithms for febrile patients are likely to require the development and incorporation of reliable diagnostic tests that provide timely diagnostic information to clinicians [37]. Unfortunately, many rapid diagnostic tests for infections related to fever management other than malaria and HIV suffer from poor performance characteristics [38], [39].

Lack of coordination among groups working on the various etiologies of febrile illness in low-resource areas has meant that sentinel studies that could provide much more comprehensive information on a wide range of responsible organisms instead have focused on only one or a small handful of etiologies. For example, a clinical trial evaluating the impact of pneumococcal conjugate vaccine on rates of *Streptococcus pneumoniae* bacteremia in a community has the potential to identify and report all bloodstream infections. Similarly, a study designed to estimate the incidence of typhoid fever to inform vaccine policy could collect acute serum along with the blood culture and, with subsequent collection of convalescent serum, would have the ability to estimate the incidence of leptospirosis and a range of other etiologic agents using conventional serologic methods [40]. However, resources for research have tended to be targeted to specific pathogens and researchers have struggled to leverage additional resources to address a broader range of organisms. Sentinel site studies seeking to understand the infectious causes of febrile illness in low-resource settings have utilized blood culture to highlight the importance of invasive bacterial and fungal infections [4], [41]. Expanding laboratory evaluations to include serologic and molecular approaches to diagnosing infections requiring specific antimicrobial management such as the bacterial zoonoses brucellosis, leptospirosis, Q fever, and the rickettsioses adds considerable value [40]. Detection of infections of public health importance such as those caused by the arboviruses dengue, Rift Valley fever, and yellow fever can inform national control programs. Since considerable etiologic overlap exists between the syndromes of fever, acute respiratory tract infection, and diarrhea [42], [43], addressing these simultaneously in integrated sentinel studies would inform enhancements in empiric treatment guidelines and improvements in the accuracy of syndrome-based disease burden estimates.

Our study had a number of limitations. While we examined a wide range of etiologies of fever, a large proportion of patients were undiagnosed suggesting that we failed to identify potentially important infections. The undiagnosed group is being investigated further using pathogen discovery approaches. Some of the diagnostic tests used in our study are less than 100% sensitive and specific and we did not test for every known pathogen. As a consequence, we probably underestimated the prevalence of some infections while misclassifying others that were falsely positive. Because a number of our diagnostic tests relied on the demonstration of a four-fold rise in antibody titer between the acute and convalescent serum sample, not all enrolled patients returned for collection of convalescent serum to have diagnoses confirmed. It follows that calculation and comparison of case fatality rate was not possible since those who died before the convalescent visit could not be confirmed cases. Incomplete diagnostic information meant that we had to extrapolate prevalence from the tested population to the untested population, potentially introducing bias. Similarly, instances of apparent infection with multiple agents were not accounted for in presentation of pie graphs. Inclusion of a well control group would have allowed the calculation of attributable fractions for individual pathogens, something that should be considered for future febrile illness research, especially in areas where malaria is endemic. Since considerable geographic variation in fever etiology is known to occur, the generalizability of our findings is uncertain.

What is needed to support an integrated approach to the syndrome of fever in resource-limited areas? First, fever should be recognized alongside pneumonia and diarrhea as a major clinical syndrome of public health importance. Achieving this is likely to require leadership from international institutions of public health and reappraisal of the way that the febrile illnesses are approached in burden of disease estimates. This could include estimating total morbidity and mortality from the syndrome of fever as a first step before attributing the associated illnesses and deaths to specific etiologies, much as is done for the other major syndromes [44], [45]. Second, efforts are needed to bring together the diverse groups and disciplines currently working on the febrile illnesses to quantify the morbidity and mortality attributable to each major etiologic agent. Such integration could be facilitated by support for research efforts that study the syndrome of fever comprehensively as well as its etiologies individually, an approach that has been modeled by studies addressing the syndromes of pediatric pneumonia and diarrhea in developing countries [46], [47]. Third, improved diagnostic services are urgently needed to establish disease burden estimates and patient management for the febrile illnesses in resource-limited areas [10]. Conventional diagnostic tests for some infections, such as leptospirosis, are complex. For example, the collection of both acute and convalescent serum samples may be required, and testing services may be available at only a few national or supra-national reference laboratories. Assays relying on convalescent samples cannot be used to estimate case fatality rates [21], [26]. Conversely, simple, rapid tests applied to acute samples may have poor performance characteristics [38]. Finally, clinical studies, including clinical trials, are needed to test and improve clinical management algorithms for febrile patients. The goal should be to target antimicrobial therapy to those who need it and to avoid inappropriate use among patients who will not benefit. In this way, patient outcomes can be improved, health resources can be conserved, and disease prevention and control efforts for febrile conditions can be rationally resourced.

## Supporting Information

Text S1 **STROBE checklist.**
(DOC)
